# Systolic Anterior Motion After Mitral Valve Repair: Echocardiographic Prediction, Surgical Prevention and Perioperative Management

**DOI:** 10.3390/jcm15186958

**Published:** 2026-09-08

**Authors:** Debora Emanuela Torre, Domenico Mangino, Giampaolo Zoffoli, Carmelo Pirri

**Affiliations:** 1 Department of Cardiac Anesthesia and Intensive Care Unit, Cardiac Surgery, Ospedale Dell’Angelo, 30174 Venice, Italy; deboraemanuela.torre@aulss3.veneto.it; 2 Cardiac Surgery Department, Ospedale Dell’Angelo, 30174 Venice, Italy; domenico.mangino@aulss3.veneto.it (D.M.); giampaolo.zoffoli@aulss3.veneto.it (G.Z.); 3 Department of Neurosciences, Institute of Human Anatomy, University of Padova, 35122 Padua, Italy

**Keywords:** systolic anterior motion, mitral valve repair, left ventricular outflow tract obstruction, mitral valve surgery, perioperative management, perioperative echocardiography

## Abstract

Systolic anterior motion (SAM) of the mitral valve remains a clinically relevant complication after mitral valve repair and may result in dynamic left ventricular outflow tract (LVOT) obstruction, SAM-associated mitral regurgitation, and hemodynamic instability. Despite advances in surgical techniques and perioperative imaging, SAM remains an important cause of difficult separation from cardiopulmonary bypass and postoperative circulatory compromise. The development of SAM is multifactorial and results from the interaction between mitral valve anatomy, ventricular geometry, surgical repair characteristics, and perioperative hemodynamic conditions. Contemporary evidence has identified several echocardiographic predictors, including excessive posterior leaflet height, elongated anterior leaflets, reduced coaptation–septal distance, a narrow mitro–aortic angle, basal septal hypertrophy, and small hyperdynamic left ventricles. Recognition of these risk factors facilitates perioperative risk assessment and pre-repair surgical planning. Transesophageal echocardiography plays a pivotal role throughout the perioperative period, enabling risk assessment before repair, early diagnosis after cardiopulmonary bypass, and guidance of therapeutic interventions. Initial treatment is based on preload optimization, afterload augmentation, withdrawal of inotropic stimulation, and heart rate control, whereas refractory cases may require surgical revision. This narrative review summarizes the current understanding of SAM after mitral valve repair, focusing on pathophysiological mechanisms, echocardiographic predictors, surgical prevention and perioperative management, with particular emphasis on the practical role of cardiac anesthesiologists and mitral valve surgeons.

## 1. Introduction

Mitral valve repair is currently considered the preferred treatment for most patients with degenerative mitral regurgitation, providing better preservation of left ventricular function, lower prosthesis-related complications, and improved long-term survival compared with valve replacement [1]. Despite significant advances in surgical techniques and perioperative imaging, systolic anterior motion (SAM) of the mitral valve remains a clinically relevant complication that may compromise the success of an otherwise satisfactory repair. SAM is characterized by anterior displacement of the mitral valve apparatus toward the interventricular septum during systole, resulting in dynamic left ventricular outflow tract (LVOT) obstruction, SAM-associated mitral regurgitation (MR), or both. The severity of clinical presentation is highly variable, ranging from an incidental echocardiographic finding without hemodynamic consequences to profound circulatory instability requiring urgent surgical revision [2,3]. In severe cases, SAM may impede separation from cardiopulmonary bypass, increase vasopressor requirements, prolong intensive care unit stay, and negatively affect postoperative outcomes. The reported incidence of SAM after mitral valve repair varies considerably according to patient characteristics, repair techniques, and diagnostic criteria, generally ranging between 2% and 10% in contemporary surgical series. Although advances in surgical planning and intraoperative transesophageal echocardiography (TEE) have improved risk recognition and preventive strategies, SAM remains an important concern during mitral valve reconstruction [4]. Historically, the pathogenesis of SAM was primarily attributed to the Venturi effect generated by accelerated systolic blood flow within the LVOT. However, advances in echocardiographic imaging and computational flow analyses have demonstrated that this explanation is incomplete. Current evidence supports a more complex mechanism in which drag forces, altered leaflet geometry, anterior displacement of the coaptation point, ventricular morphology, and perioperative loading conditions interact to produce dynamic LVOT obstruction [5,6,7]. Consequently, SAM should be viewed not merely as a postoperative complication but rather as the final manifestation of a multifactorial process involving patient anatomy, surgical repair strategy, and perioperative hemodynamics. Because most cases of clinically significant SAM develop in patients with identifiable predisposing characteristics, accurate risk stratification has become a major objective of perioperative imaging. Modern TEE allows detailed assessment of mitral valve morphology, ventricular geometry, and LVOT anatomy, enabling clinicians to identify patients at increased risk before repair and to guide both surgical planning and postoperative management [8]. Furthermore, understanding the echocardiographic predictors of SAM has facilitated the development of targeted surgical techniques designed to minimize anterior displacement of the coaptation point and reduce postoperative LVOT obstruction.

Throughout this review, SAM refers to systolic anterior displacement of the mitral leaflet or subvalvular apparatus toward the interventricular septum, irrespective of whether clinically relevant obstruction is present. SAM-associated LVOT obstruction (SAM-LVOTO) refers to dynamic outflow obstruction attributable to SAM, typically demonstrated by flow acceleration within the LVOT and a late-peaking continuous wave Doppler profile. Hemodynamically significant SAM refers to SAM associated with clinically relevant LVOT obstruction and/or SAM-associated mitral regurgitation resulting in hypotension, low cardiac output, difficulty or inability to separate from cardiopulmonary bypass, or the need for targeted therapeutic intervention. Importantly, echocardiographic SAM alone should not be considered synonymous with clinically significant LVOT obstruction, and no single Doppler gradient should be interpreted in isolation from the overall hemodynamic and anatomical context.

Previous reviews have provided important descriptions of the mechanisms and management of SAM after mitral valve repair. The present review aims to extend this literature by integrating contemporary echocardiographic predictors with a practical perioperative decision-making framework spanning pre-repair risk assessment, repair-oriented surgical prevention, immediate post-CPB diagnosis, hemodynamic management, and criteria for reconsideration of repair geometry. Particular emphasis is placed on the complementary perspectives of the cardiac anesthesiologist, echocardiographer, and mitral valve surgeon and on distinguishing evidence obtained directly in post-repair SAM from mechanistic or therapeutic evidence extrapolated from related forms of dynamic LVOT obstruction.

## 2. Materials and Methods

This narrative review was conducted to provide a comprehensive and clinically oriented overview of systolic anterior motion (SAM) following mitral valve repair, with particular emphasis on echocardiographic predictors, perioperative assessment, surgical prevention strategy, and hemodynamic management. A literature search was performed using PubMed/MEDLINE, Scopus, and Embase databases from inception to July 2026. The search strategy combined terms related to the condition and clinical setting, including “systolic anterior motion”, “SAM”, “mitral valve repair”, “mitral valve reconstruction”, “left ventricular outflow tract obstruction”, “LVOT obstruction”, “mitral valve surgery”, “echocardiographic predictors”, using appropriate Boolean combinations.

Original clinical studies, observational cohorts, surgical series, systematic and narrative reviews, clinical practice guidelines, expert consensus documents and landmark mechanistic studies published in English were considered when relevant to the predefined domains of the review. Titles and abstracts were initially assessed for relevance, followed by evaluation of the full text of potentially pertinent publications. The reference lists of key articles and previous reviews were additionally screened to identify relevant studies not retrieved through the primary database search. Studies focusing exclusively on hypertrophic cardiomyopathy were not considered as direct evidence for post-repair SAM; however, selected mechanistic or therapeutic studies from hypertrophic cardiomyopathy or other forms of dynamic LVOT obstruction were retained when they provided relevant physiologic insights not adequately addressed by the post-repair literature. Priority was given to studies specifically evaluating patients undergoing surgical mitral valve repair, particularly those reporting echocardiographic predictors, postoperative SAM, LVOT obstruction, repair-related anatomical factors, treatment strategies, or clinical outcomes. When direct evidence from post-repair populations was unavailable or limited, indirect evidence from related clinical or experimental settings was used cautiously and is identified as such in the text. Because this was a narrative review rather than a systematic review, no formal risk-of-bias assessment, quantitative evidence synthesis, or GRADE evaluation was performed. Accordingly, echocardiographic thresholds summarized in Table 1 are presented as study-derived or clinically proposed risk markers rather than universally validated cutoffs.

The selected literature was critically appraised and synthesized to provide a practical multidisciplinary framework for cardiac anesthesiologists, echocardiographers, and mitral valve surgeons.

## 3. Results

### 3.1. Mechanism of Systolic Anterior Motion

The development of SAM after mitral valve repair is the result of a complex interplay between mitral valve geometry, left ventricular morphology, intracardiac flow dynamics, and perioperative hemodynamic conditions. Although traditionally attributed to a single mechanism, SAM is now recognized as a multifactorial phenomenon in which anatomical predisposition and surgical factors interact with dynamic changes in ventricular loading conditions [6,12]. Historically, the pathophysiology of SAM was explained by the Venturi effect. According to this theory, acceleration of blood flow through the left ventricular outflow tract (LVOT) during systole generates a localized pressure drop that draws the anterior mitral leaflet toward the interventricular septum [5]. While this concept provides a plausible explanation for leaflet displacement, experimental and echocardiographic studies have demonstrated that it does not fully account for the timing and magnitude of leaflet motion observed in clinical practice [8]. Experimental, computational, and echocardiographic evidence, much of which derives from hypertrophic cardiomyopathy and mechanistic models rather than specifically from post-repair SAM, suggests that drag forces represent the predominant mechanism responsible for SAM. During ventricular systole, blood flow ejected toward the LVOT exerts a forward-directed force on the mitral leaflets [6]. When the coaptation point is displaced anteriorly, these forces act directly on the exposed ventricular surface of the leaflets, pushing them toward the interventricular septum. Once leaflet–septal contact occurs, a self-reinforcing cycle may develop, leading to progressive LVOT obstruction, further acceleration of flow, and worsening SAM. A central determinant of SAM is the anterior displacement of the mitral coaptation point [6,7,12]. In a normal mitral valve, leaflet coaptation occurs relatively posteriorly within the ventricular cavity, away from the LVOT. Conversely, excessive posterior leaflet tissue, elongated leaflets, undersized annuloplasty rings, or certain repair techniques may shift the coaptation point anteriorly, bringing the valve apparatus closer to the outflow tract and increasing susceptibility to systolic leaflet–septal interaction [7,9,12]. Left ventricular geometry also plays a critical role. Patients with small ventricular cavities, hyperdynamic systolic function, or basal septal hypertrophy exhibit a reduced distance between the mitral valve and the interventricular septum. This anatomical configuration facilitates LVOT narrowing and increases the likelihood of leaflet–septal contact. Similarly, a narrow mitro–aortic angle may orient the mitral apparatus toward the outflow tract, further predisposing to obstruction [6,9,12,13]. Importantly, SAM is not solely determined by static anatomical features. Hemodynamic conditions substantially influence its occurrence and severity. Hypovolemia, reduced afterload, tachycardia, and increased contractility all promote ventricular cavity reduction and enhance flow velocity through the LVOT, thereby exacerbating drag forces and worsening obstruction. Conversely, increased preload, higher systemic vascular resistance, and reduced contractility tend to enlarge the ventricular cavity and decrease the severity of SAM [3,4,14,15]. These observations explain why SAM frequently becomes apparent immediately after separation from cardiopulmonary bypass, when catecholamine administration, vasodilation, relative hypovolemia, and hyperdynamic ventricular function commonly coexist. Consequently, postoperative SAM should be regarded as the manifestation of a dynamic interaction between anatomical predisposition and perioperative physiology rather than a purely structural complication of mitral valve repair.

### 3.2. Echocardiographic Predictors of SAM

The ability to identify patients at increased risk of systolic anterior motion (SAM) before mitral valve repair represents one of the most important advances in contemporary perioperative echocardiography. Because SAM results from the interaction between mitral valve anatomy, ventricular geometry, and surgical repair characteristics, a comprehensive echocardiographic assessment should be performed before cardiopulmonary bypass to identify anatomical features associated with postoperative LVOT obstruction. Although no single parameter can accurately predict SAM in every patient, several echocardiographic characteristics have consistently been associated with an increased risk of postoperative obstruction. These predictors can be broadly categorized into mitral valve-related factors and left ventricular geometric factors [10,16].

For reproducibility, the principal anatomical measurements used for SAM risk assessment should be obtained during the pre-repair intraoperative TEE examination, before initiation of cardiopulmonary bypass and under reasonably stable loading conditions. In the principal post-repair prediction studies, measurements were obtained primarily from the mid-esophageal long axis view. The coaptation–septal distance is defined as the distance between the mitral leaflet coaptation point and the interventricular septum; anterior and posterior leaflet dimensions are assessed from the annular insertion toward the relevant leaflet/coaptation landmarks, while basal septal thickness and the angle between the mitral and aortic annular planes can be evaluated in the same anatomical projection. Importantly, the proposed numerical thresholds should be interpreted as study-derived risk-associated values rather than universally validated cutoffs and should therefore be integrated with the overall mitral valve and ventricular geometry rather than used as isolated predictors.

#### 3.2.1. Mitral Valve-Related Predictors

**Figure 1:**
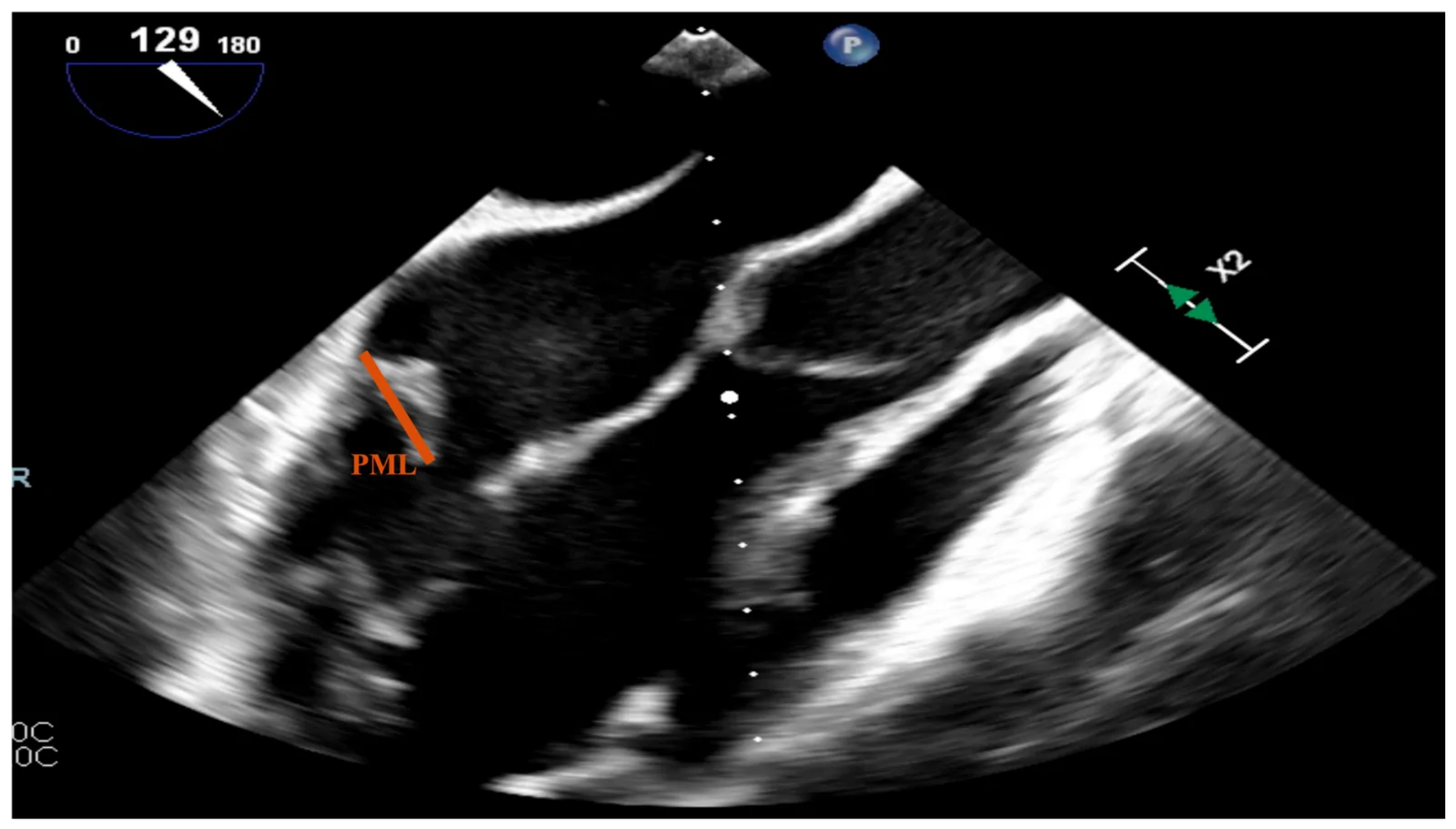
Mid-esophageal long-axis TEE view showing measurement of posterior mitral leaflet height (brown line). PML: posterior mitral leaflet.

**Figure 2:**
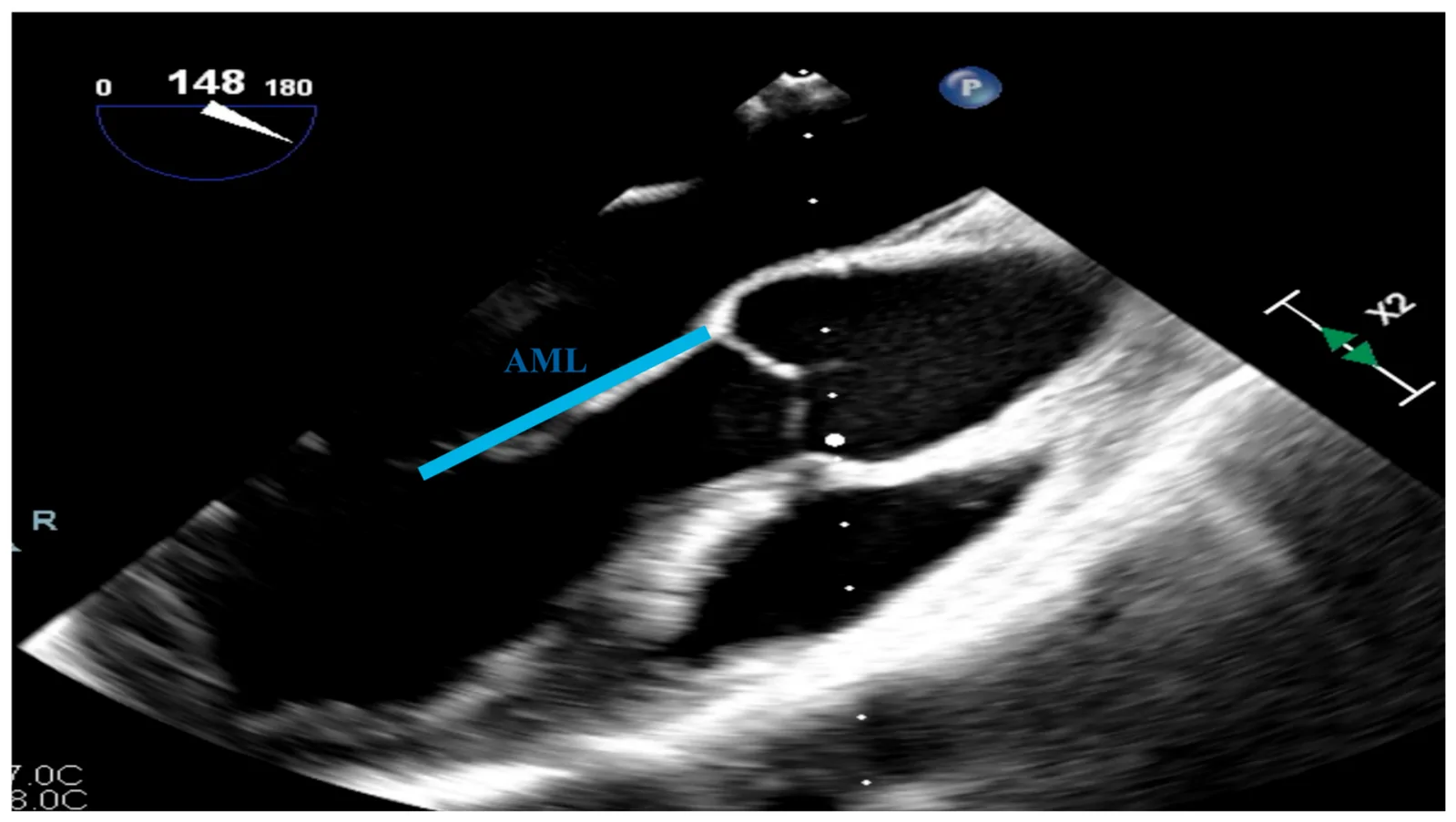
Mid-esophageal long-axis TEE view showing measurement of anterior mitral leaflet length (blue line). AML: anterior mitral leaflet.

Excess leaflet tissue is one of the most frequently recognized anatomical substrates for SAM. Patients with myxomatous degeneration, particularly those with Barlow disease, often exhibit elongated leaflets and redundant valvular tissue that predispose to anterior displacement of the coaptation point following repair. Posterior leaflet height has emerged as one of the most important predictors. Excessive posterior leaflet tissue may displace the zone of coaptation toward the LVOT, increasing the exposure of the anterior leaflet to systolic drag forces. Several studies have reported a higher incidence of SAM in patients with posterior leaflet heights exceeding 15 mm, while the risk appears particularly relevant when the height exceeds 20 mm (Figure 1). Anterior leaflet length has also been associated with postoperative SAM. An elongated anterior leaflet provides a larger surface area upon which systolic flow can act, facilitating anterior motion toward the interventricular septum. Although no universally accepted threshold exists, excessive anterior leaflet length should raise concern, particularly when combined with other geometric risk factors (Figure 2) [10,16,17]. Bileaflet prolapse represents another important predictor because both leaflets contribute to anterior displacement of the coaptation point. Consequently, patients with Barlow disease generally exhibit a greater risk of SAM than those with isolated posterior leaflet prolapse [17]. The relative proportions of the anterior and posterior leaflets may be more informative than individual measurements alone. A low anterior-to-posterior leaflet ratio reflects disproportionate posterior leaflet tissue and has been associated with increased susceptibility to SAM after repair [8,9].

#### 3.2.2. Left Ventricular and LVOT Geometry

**Figure 3:**
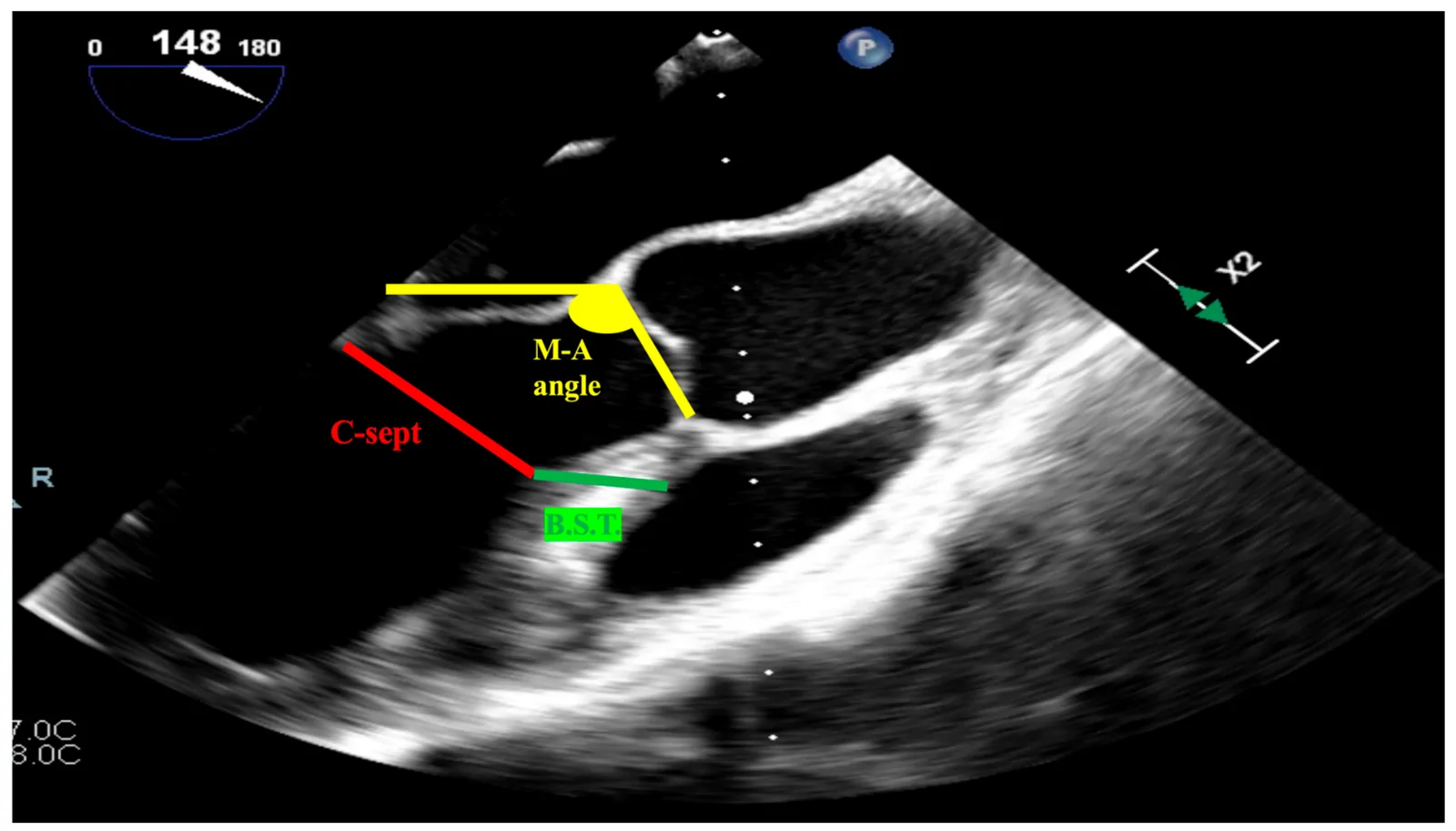
Mid-esophageal long-axis TEE view demonstrating measurement of the coaptation–septal (C-sept) distance (red line), the basal septal thickness (green line), and the mitro–aortic angle measurement (yellow line). M-A: mitro-aortic; B.S.T.: basal septal thickness.

**Figure 4:**
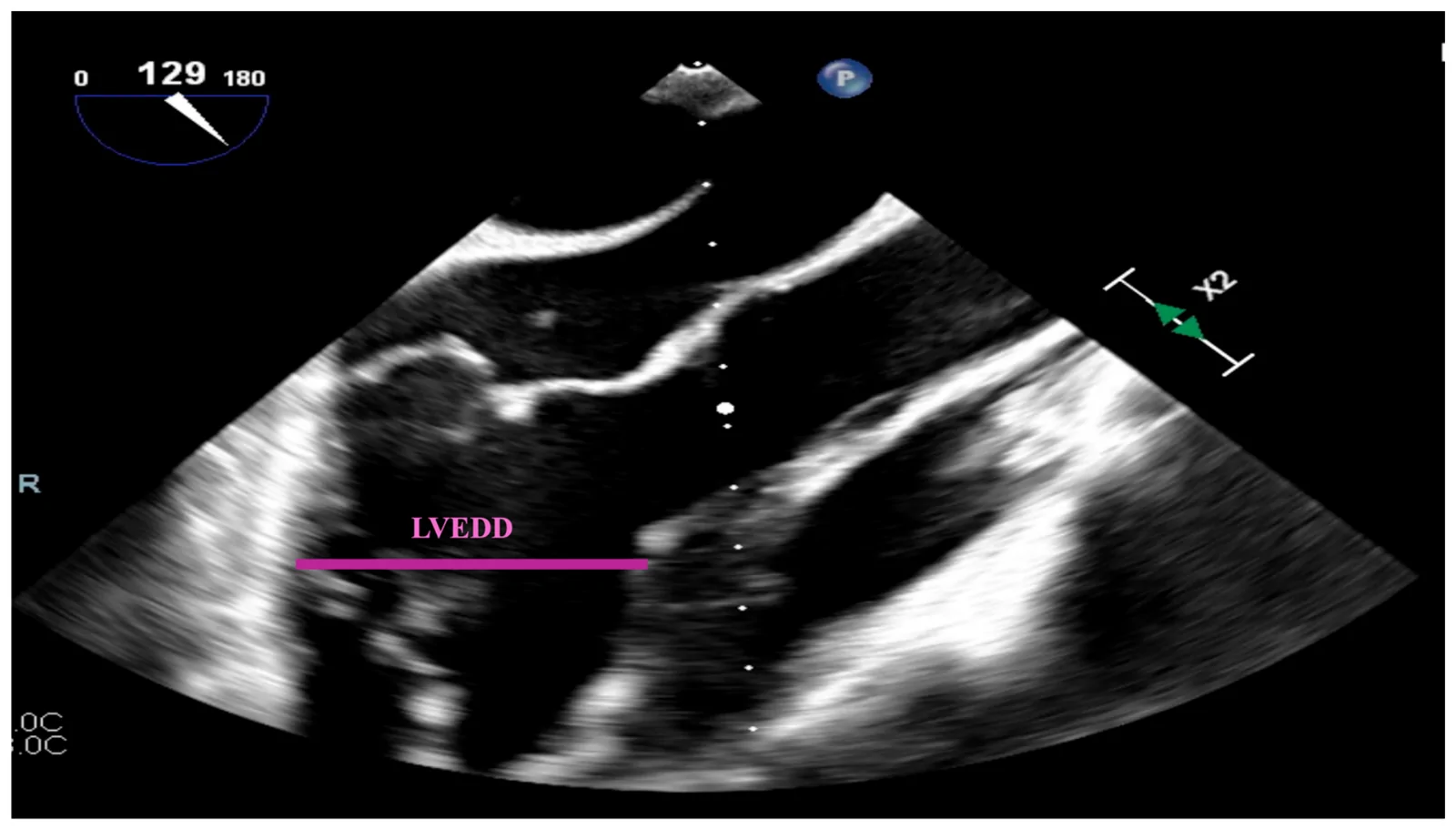
Mid-esophageal long-axis TEE view showing measurement of the left ventricular end-diastolic diameter (LVEDD) (magenta line). LVEDD: left ventricular end-diastolic diameter.

Among all echocardiographic parameters, the distance between the mitral valve coaptation point and the interventricular septum (coaptation–septal distance, C-Sept) is widely regarded as one of the strongest predictors of postoperative SAM. A reduced C-sept distance indicates that the mitral valve apparatus is positioned closer to the LVOT and therefore more likely to interact with the septum during systole. Multiple studies have demonstrated a significantly increased risk of SAM when the C-sept distance is less than 25 mm, making this measurement particularly useful during perioperative risk stratification [8,9]. The mitro–aortic angle also plays a key role. A narrow angle between the mitral and aortic annuli directs systolic flow toward the anterior mitral leaflet and favors leaflet–septal interaction. Angles below approximately 120° have been associated with an increased incidence of postoperative SAM [8,18]. Left ventricular cavity dimensions are equally important. Small ventricles with preserved or hyperdynamic systolic function are especially susceptible because systolic contraction further reduces the distance between the mitral apparatus and the septum. Patients with small end-diastolic dimensions and high ejection fractions therefore represent a classic high-risk phenotype. Basal septal hypertrophy contributes through a similar mechanism [8,15,18]. Thickening of the basal interventricular septum reduces LVOT area and decreases the available space between the septum and the mitral valve apparatus. This configuration, often encountered as a sigmoid septum in elderly patients, increases the likelihood of systolic leaflet–septal contact and may substantially increase the risk of dynamic LVOT obstruction following mitral valve repair [8,9,15]. Additional anatomical features, including a narrow LVOT and basal asymmetric septal hypertrophy, may further increase susceptibility to postoperative SAM. Although each parameter alone has limited predictive accuracy, their coexistence with established risk factors substantially increases the likelihood of postoperative LVOT obstruction (Figure 3 and Figure 4). Furthermore, acute postoperative changes in ventricular loading conditions after correction of chronic mitral regurgitation may further reduce left ventricular cavity size, amplifying the risk of SAM in anatomically predisposed patients [9,14,15].

#### 3.2.3. Practical Echocardiographic Risk Assessment

Because SAM is multifactorial, risk assessment should integrate multiple complementary echocardiographic parameters rather than rely on a single measurement (Table 1). In clinical practice, the highest-risk anatomical phenotype typically combines excessive posterior leaflet height, elongated anterior leaflet tissue, a reduced C-sept distance, a narrow mitro–aortic angle, basal septal hypertrophy, and a small hyperdynamic left ventricle. Recognition of this anatomical phenotype before mitral valve repair is essential because it allows the surgical team to adopt preventive strategies aimed at maintaining a posteriorly located coaptation point and minimizing postoperative LVOT obstruction. Equally important, identification of high-risk patients alerts the cardiac anesthesiologist to the possibility of SAM during separation from cardiopulmonary bypass and facilitates timely echocardiographic diagnosis and prompt hemodynamic intervention [10,11,14,16].

### 3.3. Intraoperative Echocardiographic Assessment

Transesophageal echocardiography (TEE) plays a pivotal role in the perioperative management of patients undergoing mitral valve repair [16,19]. Beyond confirming the mechanism and severity of mitral regurgitation, contemporary two and three-dimensional TEE enables comprehensive assessment of mitral valve morphology, ventricular geometry, and LVOT anatomy, allowing identification of patients at increased risk of postoperative SAM, optimization of surgical strategy, and immediate diagnosis of dynamic LVOT obstruction following separation from CPB. Because SAM is a dynamic phenomenon resulting from the interaction between anatomical predisposition, surgical repair characteristics, and perioperative loading conditions, echocardiographic assessment should be performed systematically both before and after mitral valve reconstruction [8,10].

#### 3.3.1. Pre-Repair Assessment

Before initiation of CPB, TEE should systematically evaluate mitral valve anatomy, left ventricular geometry, and LVOT morphology to identify patients at increased risk of postoperative SAM [19,20]. Rather than relying on a single parameter, the examination should integrate multiple anatomical findings, including leaflet morphology, coaptation geometry, ventricular dimension, septal morphology, C-sept distance, and the mitro–aortic angle, to establish the overall anatomical risk profile [4,8]. The primary objective of the pre-repair examination is not only to identify patients at risk but also to provide information that may influence the surgical strategy, including leaflet management, chordal reconstruction, annuloplasty ring selection, and adjunctive procedures aimed at maintaining a posteriorly located coaptation point [10,16].

#### 3.3.2. Post-Repair Assessment

Following separation from CPB, TEE becomes the primary tool for detecting SAM and assessing its hemodynamic significance. Examination should be performed under hemodynamic conditions that approximate the expected postoperative state, as excessive pharmacological support or profound vasodilation may either exaggerate or mask dynamic obstruction [16,19,20]. The diagnosis of SAM is established by visualization of systolic anterior displacement of the mitral leaflet apparatus toward the interventricular septum. The anterior leaflet is most commonly involved, although chordal SAM and involvement of repaired posterior leaflet tissue have also been described. Once SAM is identified, a comprehensive evaluation should be performed to determine its severity and clinical relevance. Key echocardiographic findings include the degree of leaflet–septal contact, the presence and severity of dynamic LVOT obstruction, associated mitral regurgitation, and ventricular performance [16]. Continuous-wave Doppler interrogation across the LVOT should be systematically performed. Dynamic obstruction is typically characterized by a late-peaking, dagger-shaped systolic Doppler profile. Increasing peak gradients generally correlate with greater hemodynamic significance, although management decisions should be based on the overall clinical and echocardiographic context rather than on gradient values alone. Mitral regurgitation associated with SAM usually results from incomplete leaflet coaptation caused by anterior displacement of the valve apparatus. Color Doppler assessment should determine the severity and direction of the regurgitant jet, as significant residual mitral regurgitation may influence therapeutic decisions and the need for surgical revision [6,21,22,23].

#### 3.3.3. Integrated Echocardiographic Assessment of SAM, LVOT Obstruction and Mitral Regurgitation

When SAM is detected after mitral valve repair, TEE assessment should extend beyond confirmation of anterior leaflet motion and should define the mechanism and clinical significance of the abnormality (Table 2). The presence and duration of leaflet–septal contact, the location of the reconstructed coaptation point, residual posterior leaflet height, ventricular geometry, and the relationship between the repaired valve and the LVOT should be systematically assessed. Particular attention should be paid to the mechanism of residual mitral regurgitation. SAM-associated MR results from systolic distortion of leaflet coaptation during anterior displacement of the mitral apparatus and often improves in parallel with SAM following correction of loading conditions. In contrast, persistent mitral regurgitation despite resolution of SAM should raise suspicion of an independent repair-related mechanism, including residual prolapse, leaflet restriction, inadequate coaptation, or distortion related to reconstructed valve geometry. Two-dimensional and three-dimensional imaging, color Doppler assessment of jet origin and direction, and reassessment after hemodynamic optimization are therefore complementary in distinguishing these mechanisms. Continuous-wave Doppler interrogation of the LVOT should also be interpreted cautiously because the high-velocity MR jet may contaminate or be mistaken for the LVOT signal. Dynamic LVOTO typically produces a mid-to-late systolic, late-peaking, “dagger-shaped” envelope, whereas the MR signal usually reaches higher velocities, begins earlier, and extends later into systole. Careful alignment of the Doppler cursor, localization of flow acceleration using color and pulsed-wave Doppler, and progressive sweeping between the MR jet and the LVOT may help discriminate between the two signals. Consequently, an apparently very high-Doppler-derived LVOT gradient should be verified before it is incorporated into therapeutic decision-making.

#### 3.3.4. Hemodynamic Responsiveness and Anatomical/Repair-Related Contributors

An important objective of post-repair TEE is to assess the hemodynamic responsiveness of SAM and to determine the relative contribution of anatomical and repair-related factors. SAM that improves substantially after correction of hypovolemia, vasodilation, tachycardia, or excessive inotropic stimulation can be considered predominantly hemodynamically responsive. Conversely, persistence of clinically significant SAM despite appropriate optimization of loading conditions and contractility should raise suspicion of a substantial anatomical or repair-related component, particularly in the presence of unfavorable valve geometry, excessive leaflet tissue, marked anterior displacement of the coaptation point, or inadequate correction of predisposing anatomical features. These patterns should be regarded as part of a continuum rather than as mutually exclusive entities, because postoperative SAM frequently results from the interaction between predisposing anatomy, repair geometry, and perioperative hemodynamic conditions. Serial TEE reassessment following hemodynamic optimization is therefore essential to determine the predominant mechanism and guide subsequent management [2,14,16].

### 3.4. Surgical Strategies for Prevention and Correction of SAM

Prevention remains the most effective strategy for managing systolic anterior motion after mitral valve repair. Because postoperative SAM is largely determined by the interaction between native anatomy and repair geometry, surgical planning should aim to preserve a posteriorly located coaptation point and maintain an adequate distance between the mitral valve apparatus and the LVOT. Recognition of high-risk anatomical features during preoperative and intraoperative echocardiographic assessment allows the surgeon to modify repair techniques accordingly, thereby minimizing the likelihood of postoperative obstruction [24,25,26], [Table 3].

#### 3.4.1. Posterior Leaflet Height Reduction

Excessive posterior leaflet tissue is one of the most important anatomical contributors to SAM. A tall posterior leaflet may displace the coaptation point anteriorly, increasing the exposure of the anterior mitral leaflet to systolic drag forces and promoting leaflet–septal interaction [8,11]. Several surgical techniques have been developed to reduce posterior leaflet height and restore a more physiological coaptation geometry. These include sliding leaflet plasty, folding plasty, and selective leaflet resection. By decreasing posterior leaflet height, these procedures facilitate posterior displacement of the coaptation zone and reduce the risk of LVOT obstruction [25,27,28,29]. Among these approaches, sliding plasty remains one of the most widely adopted techniques in patients with excessive posterior leaflet tissue and has consistently been associated with lower rates of postoperative SAM [30].

#### 3.4.2. Chordal-Based Repair Strategies

Over the last two decades, artificial chordal implantation has become a cornerstone of mitral valve repair [31]. Chordal replacement preserves leaflet mobility while avoiding excessive leaflet resection and may contribute to a more physiological valve configuration. However, excessive leaflet tissue may predispose to SAM despite successful chordal reconstruction [8,32]. Consequently, neo-chordal implantation alone may be insufficient in patients with marked leaflet redundancy, and concomitant leaflet height reduction may be necessary. Careful adjustment of neo-chordal length is also essential, as an excessively tall, reconstructed leaflet may contribute to anterior displacement of the coaptation point [17].

#### 3.4.3. Annuloplasty Ring Selection

Annuloplasty ring implantation is a fundamental component of contemporary mitral valve repair [33]. Nevertheless, inappropriate ring sizing may contribute to SAM by altering mitral valve geometry. Undersized annuloplasty rings may reduce annular dimensions excessively and promote anterior displacement of the coaptation point. Conversely, appropriate ring sizing helps maintain physiological leaflet relationships and reduces the likelihood of postoperative LVOT obstruction. For this reason, ring selection should always be individualized according to leaflet dimensions, annular geometry, and ventricular anatomy rather than based on standardized sizing strategies alone [34].

#### 3.4.4. Edge-to-Edge Repair as Rescue Strategy

When significant SAM persists despite optimization of repair geometry and hemodynamic conditions, edge-to-edge repair may provide an effective surgical solution. By creating a double orifice valve configuration, the edge-to-edge technique can shift the coaptation point posteriorly and reduce anterior leaflet excursion toward the interventricular septum. Although not routinely employed as a preventive measure, this approach has demonstrated efficacy as a rescue strategy in selected patients with persistent postoperative SAM [35,36,37]. Nevertheless, the edge-to-edge technique alters mitral valve geometry by creating a double orifice configuration and may increase trans-mitral pressure gradients. Therefore, following edge-to-edge repair, intraoperative TEE should systematically reassess residual mitral regurgitation, leaflet mobility, and peak and mean trans-mitral gradients to exclude clinically relevant iatrogenic mitral stenosis. Although mildly increased gradients may be observed after edge-to-edge repair, markedly elevated gradients should prompt careful reassessment of the adequacy of the repair [37,38].

#### 3.4.5. Concomitant Septal Reduction Procedures

In selected patients, particularly those with marked basal septal hypertrophy, septal morphology may represent a major contributor to postoperative LVOT obstruction. When significant septal hypertrophy is identified preoperatively, concomitant septal myectomy may be considered during mitral valve surgery [8]. Enlargement of the LVOT increases the distance between the mitral valve apparatus and the interventricular septum, thereby reducing the likelihood of leaflet–septal contact. Although septal reduction is required only in a minority of patients, careful preoperative recognition of unfavorable septal anatomy is essential because medical management alone may be insufficient when obstruction is predominantly anatomy-driven [25,39]. Evidence for septal reduction in isolated post-repair SAM is limited largely to selected surgical reports and should not be extrapolated from obstructive HCM without considering the underlying repair geometry.

### 3.5. Perioperative Hemodynamic Management

**Figure 5:**
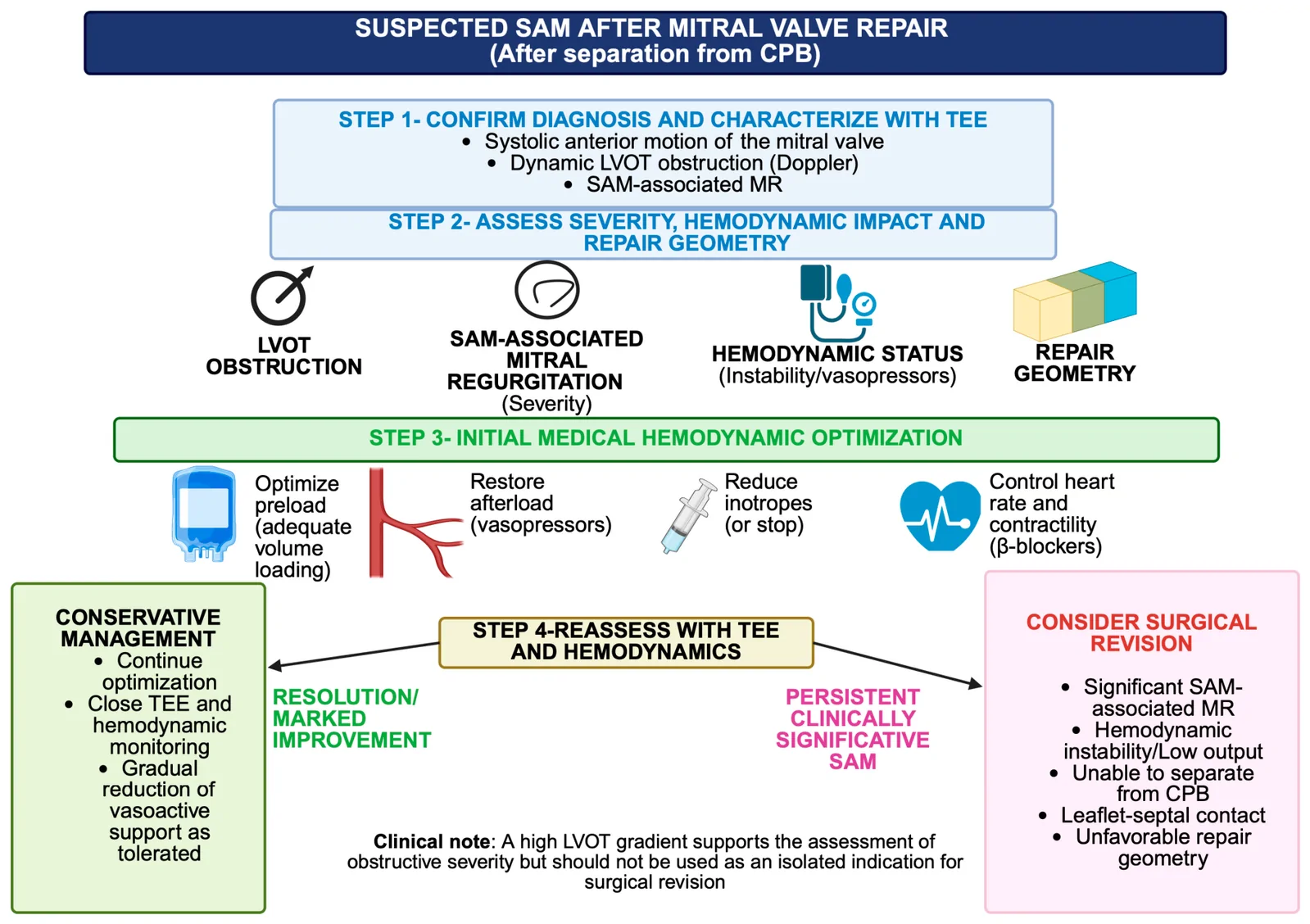
Practical perioperative algorithm for the diagnosis and management of SAM after mitral valve repair. CPB: cardiopulmonary bypass; MR: mitral regurgitation; TEE: transesophageal echocardiography; LVOT: left ventricular outflow tract; SAM: systolic anterior motion. Created in BioRender. Pirri, C. https://BioRender.com/8bvcsog (accessed on 25 August 2026).

The management of SAM after mitral valve repair is primarily guided by its underlying pathophysiology. Because the severity of LVOT obstruction is highly dependent on ventricular loading conditions and contractile state, hemodynamic optimization represents the cornerstone of initial treatment. In many cases, prompt recognition and appropriate medical management can successfully reverse SAM and avoid surgical revision. The primary therapeutic objective is to reduce the forces promoting anterior leaflet displacement while simultaneously increasing the distance between the mitral valve apparatus and the interventricular septum. This is achieved through restoration of preload, augmentation of afterload, reduction in contractility, and control of heart rate (Figure 5) [4,14].

#### 3.5.1. Optimization of Preload

Hypovolemia is a well-recognized precipitating factor for SAM because reduction in ventricular filling decreases left ventricular cavity size and promotes leaflet–septal interaction [10]. Relative hypovolemia is particularly common following separation from cardiopulmonary bypass due to vasodilation, capillary leak, and redistribution of intravascular volume [40]. Volume administration therefore represents one of the first therapeutic interventions when SAM is identified. Increasing preload enlarges ventricular dimensions, increases the distance between the mitral valve and the interventricular septum, and may substantially reduce the severity of dynamic obstruction. However, excessive volume loading should be avoided, particularly in patients with impaired ventricular compliance or significant right ventricular dysfunction. Echocardiographic assessment remains essential to guide individualized fluid administration [39,41].

#### 3.5.2. Augmentation of Afterload

Reduced systemic vascular resistance facilitates ventricular emptying and increases flow acceleration through the LVOT, thereby exacerbating SAM. Consequently, restoration of afterload is a key component of treatment. Pure vasoconstrictors are generally preferred because they increase systemic vascular resistance without directly stimulating myocardial contractility [2,42,43,44]. Phenylephrine is particularly attractive from a physiological standpoint because it increases afterload, reduces heart rate through reflex vagal activation, and may attenuate dynamic obstruction [2,42,43,44,45].

Norepinephrine is frequently used in clinical practice owing to its potent vasopressor effects and widespread availability. Although its mild β-adrenergic activity may theoretically increase contractility, restoration of arterial pressure and afterload often results in a net improvement in LVOT obstruction [42,43,44].

Evidence supporting vasopressin is primarily derived from other forms of dynamic LVOTO and vasodilatory states; its use in post-repair SAM therefore remains physiologically plausible rather than specifically validated [46,47].

#### 3.5.3. Reduction in Contractility

Hyperdynamic ventricular contraction is a major determinant of SAM. Enhanced systolic shortening reduces ventricular cavity size and increases drag forces acting on the mitral leaflets [8,48]. Whenever clinically feasible, inotropic agents should therefore be reduced or discontinued. Catecholamines administered during separation from CPB may precipitate or exacerbate dynamic LVOT obstruction and should be reassessed whenever SAM is detected [49]. In selected patients, β-adrenergic blockade may be highly effective. By reducing contractility and prolonging diastolic filling, beta-blockers decrease the severity of SAM and improve ventricular filling. Short-acting agents such as esmolol are particularly useful because they allow rapid titration and immediate reversal if hemodynamic deterioration occurs [43,50,51].

#### 3.5.4. Heart Rate Control

Tachycardia contributes to SAM through multiple mechanisms. Shortened diastolic filling reduces preload, while increased contractile stimulation enhances systolic flow acceleration within the LVOT. Maintenance of sinus rhythm and avoidance of excessive heart rates are therefore important therapeutic objectives. Beta-blockers may simultaneously reduce heart rate and contractility, providing a dual benefit in the management of SAM [50,51].

#### 3.5.5. Hemodynamic Reassessment

Because SAM is highly dynamic, repeated echocardiographic reassessment is mandatory following any therapeutic intervention [19,20]. Changes in preload, afterload, contractility, or heart rate may rapidly modify the severity of obstruction and alter the need for additional treatment [4,14,42]. The response to hemodynamic optimization provides important diagnostic information. Resolution or substantial improvement of SAM following correction of loading conditions indicates a predominantly hemodynamically responsive component [2,4,42]. In contrast, persistence of clinically significant SAM and/or LVOT obstruction despite appropriate optimization should raise suspicion of a substantial anatomical or repair-related component and prompt careful reassessment of repair geometry and consideration of surgical revision when appropriate [2,4,10]. For this reason, management should not focus exclusively on the LVOT gradient. Instead, therapeutic decisions should integrate multiple factors, including the severity of SAM, degree of mitral regurgitation, ventricular performance, hemodynamic stability, and response to medical therapy [4,19,20,52].

### 3.6. When Medical Therapy Fails: Indications for Surgical Revision

Although most cases of postoperative SAM respond to optimization of preload, afterload, contractility, and heart rate, a subset of patients exhibit persistent hemodynamically significant obstruction despite appropriate medical management [2,4,14,42]. In these situations, surgical revision should be considered without unnecessary delay [4,42,52]. The decision to proceed with reintervention should not be based on a single echocardiographic parameter but rather on the integration of clinical, hemodynamic, and imaging findings [4,19,20,42].

Importantly, the decision to return to cardiopulmonary bypass (CPB) should not be based on a single echocardiographic parameter or an isolated LVOT gradient. Although a peak LVOT gradient > 50 mmHg has been incorporated into previously published management algorithms for post-repair SAM, this threshold should be regarded as a marker of potentially important dynamic obstruction rather than as an independently validated or universal indication for surgical revision. Instead, decision-making should integrate the persistence and severity of SAM, the degree of associated mitral regurgitation, the patient’s hemodynamic status, the ability to separate from CPB, the response to correction of loading conditions and withdrawal of inotropic stimulation, and the geometry of the repaired mitral valve [4,19,20,42,52]. Accordingly, return to CPB should be strongly considered when clinically significant SAM persists despite adequate preload restoration, afterload augmentation, reduction or discontinuation of inotropic stimulation, and appropriate control of heart rate and contractility, particularly when accompanied by moderate-to-severe SAM-associated mitral regurgitation, persistent hypotension or low cardiac output, recurrent hemodynamic deterioration, or inability to separate safely from CPB. Echocardiographic evidence of sustained leaflet–septal contact, marked anterior displacement of the coaptation point, excessive residual posterior leaflet height, unfavorable leaflet proportions, or significant septal encroachment on the LVOT further supports the presence of a substantial anatomical or repair-related component that is unlikely to resolve with additional hemodynamic manipulation alone [8,9,10,15,42]. Because postoperative SAM usually results from a continuum of interactions among native anatomy, repair geometry, ventricular morphology, and loading conditions, a strict distinction between “functional” and “anatomical” SAM may be overly simplistic. A more clinically useful approach is to distinguish “predominantly hemodynamically responsive SAM”, which improves substantially after correction of reversible physiological factors, from “persistent SAM with significant anatomical or repair-related component” in which obstruction and/or mitral regurgitation remain clinically relevant despite appropriate hemodynamic optimization. This distinction should be established through serial TEE assessment under progressively optimized hemodynamic conditions [2,4,8,9,10,14,15,19,20,42]. When surgical revision is required, the corrective strategy should be tailored to the mechanism identified by echocardiography and direct surgical inspection. Excessive posterior leaflet height may require additional leaflet-height reduction or resection, whereas marked anterior displacement of the coaptation point may necessitate modification of leaflet geometry, chordal length, or annuloplasty strategy [25,27,28,29,30,34]. In selected patients, edge-to-edge repair may effectively reduce anterior leaflet excursion and relocate the coaptation zone posteriorly; however, post-repair TEE should confirm satisfactory valve function and exclude an excessive trans-mitral gradient or clinically relevant iatrogenic mitral stenosis. In patients with substantial basal septal hypertrophy contributing to persistent LVOT obstruction, septal reduction may occasionally be considered, although evidence supporting this approach specifically in post-repair SAM remains limited and patient selection should therefore be individualized [35,36,37]. Mitral valve replacement should remain a last-resort bailout strategy but may occasionally be necessary when clinically significant SAM, LVOT obstruction, or mitral regurgitation cannot be adequately corrected despite hemodynamic optimization and one or more attempts at repair revision [2]. Whenever feasible, however, mechanism-directed revision of the repair should be preferred in order to preserve the benefits of mitral valve reconstruction. Serial intraoperative TEE reassessment is fundamental throughout this process. Resolution or marked reduction in SAM, LVOT obstruction, and associated mitral regurgitation following hemodynamic optimization generally supports continued conservative management, whereas persistent obstruction associated with unfavorable repair geometry or hemodynamic compromise should prompt early multidisciplinary discussion between cardiac anesthesiologists, echocardiographers, and surgeons regarding return to CPB. The magnitude of the LVOT gradient should therefore be interpreted as one component of an integrated anatomical and hemodynamic assessment rather than as an isolated trigger for surgical intervention [2,4,11,35,36,37,38,39,42,52].

## 4. Discussion

Despite significant advances in mitral valve repair techniques and perioperative imaging, systolic anterior motion (SAM) remains an important cause of postoperative hemodynamic instability [3,4,14]. The contemporary understanding of SAM has evolved considerably from the traditional concept of a purely flow-mediated phenomenon to a more comprehensive model integrating valve geometry, ventricular morphology, surgical repair characteristics, and dynamic loading conditions [7,12,14,22]. One of the most relevant findings emerging from the available literature is that clinically significant postoperative SAM is frequently associated with identifiable predisposing anatomical and repair-related features, although its occurrence and severity remain influenced by dynamic perioperative loading conditions [8,9,10]. Most patients who develop clinically significant obstruction exhibit identifiable preoperative anatomical features, including excessive posterior leaflet height, reduced coaptation–septal distance, small hyperdynamic ventricles, basal septal hypertrophy, or a combination of these factors [8,9,10,18]. Consequently, meticulous perioperative echocardiographic assessment has become essential not only for diagnosis but also for risk stratification and surgical planning [16,19,20]. Another important concept is the dynamic nature of SAM [2,14,42]. Unlike fixed forms of LVOT obstruction, the severity of SAM may change rapidly in response to alterations in preload, afterload, contractility, and heart rate [2,4,42]. This explains why patients with apparently satisfactory surgical repairs may develop significant obstruction immediately after separation from CPB, when vasodilation, catecholamine administration, and relative hypovolemia frequently coexist. Recognition of this pathophysiological interaction is fundamental because many cases can be successfully managed through targeted hemodynamic optimization without requiring surgical revision [2,4,42,52]. The present review also highlights the central role of transesophageal echocardiography throughout the perioperative period. Beyond confirming the presence of SAM, TEE provides critical information regarding its underlying mechanism, hemodynamic significance, and response to treatment. In this context, echocardiography should not be viewed as a purely diagnostic tool but rather as an integral component of decision-making that guides both medical and surgical management [19,20]. From a surgical perspective, contemporary repair strategies designed to optimize leaflet geometry and maintain posterior coaptation may reduce the risk of postoperative SAM in appropriately selected patients [11,17,26]. Greater understanding of the relationship between leaflet geometry and LVOT obstruction has encouraged the adoption of tailored repair techniques aimed at maintaining a posteriorly located coaptation point and minimizing leaflet–septal interaction [26,27,30]. Nevertheless, no single surgical approach completely eliminates the risk of SAM, emphasizing the importance of individualized treatment based on patient-specific anatomy [4,10,17]. Finally, effective management of SAM requires close collaboration between cardiac surgeons and cardiac anesthesiologists. Successful outcomes depend on the integration of echocardiographic findings, surgical expertise, and hemodynamic management [4,19,20,42]. This multidisciplinary approach is particularly important in assessing the relative contribution of hemodynamic responsiveness and anatomical or repair-related factors, because these components frequently coexist and have important implications for subsequent management [2,4,42]. Taken together, the available evidence supports a perioperative strategy centered on early identification of high-risk anatomy, systematic intraoperative echocardiographic assessment, prompt hemodynamic optimization, and timely surgical intervention when necessary. Such an approach offers the greatest opportunity to minimize morbidity and maximize the durability and success of mitral valve repair.

### Limitations and Heterogeneity of the Available Evidence

The evidence supporting the prediction, prevention, and management of SAM after mitral valve repair has several important limitations. Most available data derive from retrospective or observational surgical series, frequently conducted at single high-volume centers, and randomized or prospectively validated evidence is largely lacking. Moreover, several landmark studies were performed in the context of surgical techniques and perioperative practice that have subsequently evolved, potentially limiting the generalizability of some findings to contemporary mitral valve repair. Considerable heterogeneity also exists in the definition of SAM and its clinical significance. Some studies define SAM primarily as an echocardiographic finding, whereas others focus on SAM associated with dynamic LVOT obstruction, mitral regurgitation, or hemodynamic compromise. Similarly, the hemodynamic conditions under which postoperative echocardiographic assessment is performed are not standardized across studies and may substantially influence the presence and severity of dynamic obstruction. Echocardiographic risk markers should therefore be interpreted cautiously. Several commonly cited thresholds, including coaptation–septal distance, posterior leaflet height, mitro–aortic angle, basal septal thickness, and LV dimensions, were derived from observational cohorts and have not been uniformly externally validated across different populations and repair techniques. These values should consequently be regarded as risk-associated markers that contribute to an integrated anatomical assessment rather than as universal standalone cutoffs. The evidence supporting therapeutic strategies is similarly heterogeneous. Hemodynamic optimization and mechanism-directed surgical revision are supported by consistent physiological rationale and observational clinical experience; however, comparative evidence between specific pharmacological or surgical strategies remains limited. In addition, some mechanistic and therapeutic concepts applied to post-repair SAM are extrapolated from hypertrophic cardiomyopathy or other forms of dynamic LVOT obstruction and should therefore be interpreted as indirect supportive evidence rather than as direct evidence specific to mitral valve repair.

From an evidence perspective, the most consistent findings concern the association between unfavorable post-repair geometry and SAM susceptibility and the dynamic dependence of obstruction on loading conditions. In contrast, the precise predictive performance of individual echocardiographic thresholds, the superiority of specific surgical preventive techniques, and the comparative effectiveness of pharmacological strategies remain less firmly established.

Accordingly, the recommendations summarized in this review should be considered within a multidisciplinary and individualized decision-making framework. Future prospective multicenter studies using standardized definitions of SAM, SAM-associated LVOT obstruction and clinically significant SAM, together with standardized echocardiographic acquisition and reporting, are needed to improve risk prediction and establish more robust management criteria.

## 5. Conclusions

SAM remains one of the most important and potentially reversible complications following mitral valve repair. Contemporary evidence indicates that SAM is not merely a postoperative event but rather the result of a complex interaction between mitral valve anatomy, ventricular geometry, surgical repair characteristics, and perioperative hemodynamic conditions. Comprehensive transesophageal echocardiography is central to modern SAM management, enabling perioperative and pre-repair risk assessment, intraoperative diagnosis, and guidance of therapeutic interventions. Among the various echocardiographic predictors, reduced coaptation–septal distance, excessive posterior leaflet height, a small hyperdynamic left ventricle, and basal septal hypertrophy appear to be the most clinically relevant risk factors for postoperative obstruction. Most cases can be successfully managed through prompt hemodynamic optimization based on preload augmentation, afterload restoration, reduction in inotropic stimulation, and heart rate control. However, persistent clinically significant SAM with a substantial anatomical or repair-related component requires timely recognition and, in selected patients, surgical revision. Close collaboration between cardiac anesthesiologists, echocardiographers, and cardiac surgeons is therefore essential to achieve optimal outcomes.

## 6. Future Directions

Emerging computational and artificial intelligence-based approaches may eventually complement conventional echocardiographic assessment for prediction of SAM after mitral valve repair. In particular, patient-specific computational models offer promising potential for future applications in estimating post-repair SAM risk and simulating the potential effects of different repair configurations [53,54,55,56,57,58,59]. However, available studies remain limited, largely retrospective, and have not yet established externally validated tools for routine perioperative decision-making. Similarly, automated three-dimensional valve segmentation and machine-learning approaches are increasingly able to characterize mitral anatomy, but SAM-specific clinical prediction remains investigational [58]. Future studies should therefore focus on external validation, integration of anatomical and hemodynamic variables, and prospective evaluation of whether these technologies improve surgical planning or patient outcomes beyond expert echocardiographic assessment.

## Figures and Tables

**Table 1:** Echocardiographic predictors of systolic anterior motion after mitral valve repair. AML: anterior mitral leaflet; PML: posterior mitral leaflet; LV: left ventricle; LVEDD: left ventricular end-diastolic diameter; LVOT: left ventricular outflow tract; LVEF: left ventricular ejection fraction.

Parameter	Risk-Associated Value	Interpretation	Evidence Basis
Coaptation–septal distance (C-sept)	<25 mm	Reduced mitral–septal separation facilitates leaflet–septal contact	Direct post-repair cohort evidence [8,9]
Posterior leaflet height (PML height)	>15 mm (particularly >20 mm)	Anterior displacement of the coaptation point toward the LVOT	Direct post-repair cohort evidence [8]
Anterior mitral leaflet length (AML)	No universally validated cutoff	Increased leaflet surface exposed to systolic drag forces	Supportive post-repair evidence [8,9,10]
AML/PML ratio	Lower anterior/posterior leaflet height ratio; no universal cutoff	Relative excess of posterior leaflet tissue promotes anterior coaptation shift	Direct post-repair observational evidence [9,10]
Mitro–aortic angle	<120°	Directs systolic flow toward the anterior leaflet and LVOT	Direct post-repair cohort evidence [8]
Basal septal thickness	>=15 mm	LVOT narrowing and reduced mitral–septal distance	Direct post-repair cohort evidence [8]
LV end-diastolic diameter (LVEDD)	<45 mm	Small ventricular cavity predisposes to dynamic obstruction	Direct post-repair cohort evidence [8]
Hyperdynamic LV function	No universally validated LVEF cutoff	Hyperdynamic contraction increases drag forces	Supportive post-repair evidence [10,11]
Bi-leaflet prolapse	Presence	Excess leaflet tissue and anterior coaptation displacement	Direct post-repair observational evidence [10]

The threshold summarized should not be interpreted as universal diagnostic cutoffs. Values reported for C-sept distance, posterior leaflet height, mitro–aortic angle, basal septal thickness and LVEDD were derived from observational cohorts of patients undergoing mitral valve repair and identify anatomical characteristics associated with increased risk. For other parameters, available evidence supports an association with SAM susceptibility but does not establish a reproducible numerical threshold.

**Table 2:** Practical perioperative transesophageal echocardiography checklist for the assessment of SAM after mitral valve repair. AML: anterior mitral leaflet; PML: posterior mitral leaflet; CPB: cardiopulmonary bypass; SAM: systolic anterior motion; LVOT: left ventricular outflow tract; C-sept: coaptation–septal distance.

Assessment Phase	Key Question	Clinical Relevance
Pre-repair	Is the patient anatomically predisposed to SAM?	Identifies high-risk anatomy requiring preventive surgical planning
Pre-repair	What is the C-Sept?	Reduced distance (<25 mm) is strongly associated with postoperative SAM
Pre-repair	Are AML and PML dimensions excessive?	Excess leaflet tissue may shift the coaptation point anteriorly
Pre-repair	Is basal septal hypertrophy present?	May reduce LVOT area and facilitate obstruction
Pre-repair	Is the mitro–aortic angle reduced?	Narrow angles increase the likelihood of leaflet–septal interaction
Post-repair	Is the coaptation point displaced anteriorly?	Suggests an increased risk of postoperative SAM
Post-repair	Is SAM present?	Establishes the diagnosis
Post-repair	Is dynamic LVOT obstruction present?	Determines the hemodynamic significance of SAM
Post-repair	Is residual mitral regurgitation present?	Assesses repair adequacy and SAM severity
Post-repair	Does SAM improve after hemodynamic optimization?	Assesses hemodynamic responsiveness and helps identify a persistent anatomical or repair-related component
Post-repair	Is surgical revision required?	Guides decision-making when medical therapy fails

**Table 3:** Surgical strategies for prevention and correction of postoperative SAM. AML: anterior mitral leaflet; PML: posterior mitral leaflet; SAM: systolic anterior motion; LVOT: left ventricular outflow tract.

Surgical Strategy	Mechanism	Expected Effect on SAM
Sliding leaflet plasty	Reduces PML height	↓ SAM risk
Folding plasty	Decreases leaflet redundancy	↓ SAM risk
Limited posterior leaflet resection	Posteriorizes coaptation	↓ SAM risk
Artificial chordae	Restores leaflet geometry; effect depends on neochordal length and residual leaflet height	May contribute to SAM prevention when appropriately tailored; not independently protective in the presence of marked leaflet redundancy
Appropriate ring sizing	Preserves mitral geometry	↓ SAM risk
Edge-to-edge repair	Posterior displacement of coaptation	Rescue therapy
Septal myectomy	Enlarges LVOT	↓ LVOT obstruction

Down arrow (↓) = decrease.

## Data Availability

No new data were created or analyzed in this study. Data sharing is not applicable to this article.

## Abbreviations

The following abbreviations are used in this manuscript:

SAM	Systolic anterior motion
LVOT	Left ventricular outflow tract
AML	Anterior mitral leaflet
PML	Posterior mitral leaflet
CPB	Cardiopulmonary bypass
